# Salidroside Represses the Viability of Mouse Sertoli Cell Line (TM4 Cells) via Repression of *Ldha*-Dependent Glycolysis

**DOI:** 10.3390/molecules31193575

**Published:** 2026-10-08

**Authors:** Xinran Wang, Ruixue Huang, Gan Yang, Yuxia He, Shi Yin

**Affiliations:** College of Animal & Veterinary Sciences, Southwest Minzu University, Chengdu 610041, China; 13050382873@163.com (X.W.); huangruixue2022@163.com (R.H.); 18288246608@163.com (G.Y.); 18987366903@163.com (Y.H.)

**Keywords:** salidroside, Sertoli cells, cell viability, glycolysis, *Ldha*

## Abstract

Salidroside is a bioactive compound extracted from the traditional Chinese medicinal plant *Rhodiola rosea* that exerts differential regulatory effects on cell viability depending on cell type and physiological state. Sertoli cells are important testicular somatic cells that play multiple roles in nourishing, protecting, and supporting spermatogenic cells. Currently, the regulatory effects and underlying mechanisms of salidroside on Sertoli cells remain unclear. This study investigated the effects of different concentrations of salidroside on the viability and regulatory mechanisms of TM4 cells (mouse Sertoli cell line). The results showed that within the range of 25–600 μM, cell viability decreased with increasing salidroside concentration. Treatment with 600 μM salidroside inhibited proliferation, promoted apoptosis, and suppressed the expression of key genes related to Sertoli cell function. Transcriptome sequencing revealed that several glycolysis-related genes, including *Ldha*, *Pfkl*, *Eno3*, and *Eno1b*, were significantly downregulated in salidroside-treated cells, accompanied by marked reductions in key glycolytic metabolites, including fructose-1,6-diphosphate (FDP), pyruvate, lactic acid, and ATP. Notably, overexpression of lactate dehydrogenase A (*Ldha*) rescued cell viability and proliferation, suppressed apoptosis, elevated both intracellular and extracellular lactate and ATP levels, and partially restored functional gene expression in 600 μM salidroside-treated cells. Overall, this study provides hitherto undocumented evidence of the regulatory effects and mechanisms of salidroside on TM4 cells, identifying the *Ldha*–glycolytic axis as an in vitro cellular target of high-dose salidroside in Sertoli cells, which may inform future in vivo reproductive safety profiling pending pharmacokinetic validation.

## 1. Introduction

Sertoli cells (SCs) are the sole somatic cells within the seminiferous epithelium of the mammalian testis that directly contact germ cells [1,2]. SCs ensure the proper progression of spermatogenesis through various mechanisms, such as paracrine signaling, blood–testis barrier (BTB) formation, and metabolic regulation. Studies have demonstrated that factors secreted by SCs, such as platelet-derived growth factor (PDGF), glial cell line-derived neurotrophic factor (GDNF), and stem cell factor (SCF), regulate the proliferation and differentiation of spermatogonia and maintain the self-renewal capacity of spermatogonial stem cells [3,4,5]. Adjacent SCs, as well as junctions between SCs and germ cells, form the blood–testis barrier (BTB) via tight junctions. The BTB establishes an immune-privileged microenvironment within the seminiferous tubules, restricting the passage of harmful substances and thereby maintaining a stable milieu essential for spermatogenesis [6,7]. Furthermore, SCs exhibit metabolic characteristics analogous to the Warburg effect in tumor cells, preferentially undergoing glycolysis even under aerobic conditions [8,9]. The lactate produced via lactate dehydrogenase serves not only as an energy source for SCs themselves but also modulates critical physiological processes in developing germ cells [10,11].

*Rhodiola rosea*, a perennial herbaceous plant belonging to the genus *Rhodiola* family, is a widely used medicinal herb that grows in high-altitude regions at elevations of 1800–2000 m [12,13]. Salidroside (SAL), a bioactive compound derived from *Rhodiola rosea*, reportedly possesses significant pharmacological properties including antioxidant, anti-aging, and anti-inflammatory effects. Studies have demonstrated that SAL delays oocyte aging by activating the MAPK signaling pathway to regulate oxidative stress [14,15]. In addition, it reverses premature senescence in skin fibroblasts by enhancing cellular viability and suppressing senescence-associated enzymatic activity [16]. SAL also exhibits anti-inflammatory action by inhibiting nuclear factor kappa-B (NF-κB) and activating the ERK/MAPKs pathway, thereby modulating macrophage-derived pro-inflammatory cytokine production [17].

In addition to its well-documented pharmacological benefits, SAL has been shown to exhibit certain toxicological effects on various cell types through different mechanisms. Studies have demonstrated that under hypoxic conditions, SAL triggers autophagy in pulmonary artery smooth muscle cells via the AMPKα1-mTOR-ULK1 signaling axis, leading to significant suppression of cell proliferation [18]. SAL also inhibits endothelial cell apoptosis induced by oxidized low-density lipoprotein by downregulating microRNA-133a [19]. SAL has been demonstrated to selectively target and kill tumor cells by inhibiting the glycolytic pathway. For instance, in human gastric cancer cells, SAL was found to suppress glycolysis by downregulating key glycolytic enzymes, including pyruvate kinase isoenzyme M2 (PKM2), enolase 1 (ENO1), and glucose transporter 1 (GLUT1), thereby promoting tumor cell apoptosis [20]. Similarly, in ovarian cancer cells, SAL has been found to exert its antitumor effects by inhibiting the STAT3/c-Myc pathway-mediated glycolysis [21]. Interestingly, SCs exhibit aerobic glycolysis similar to tumor cells, relying primarily on glycolysis rather than oxidative phosphorylation for energy production. Although crucial for maintaining the spermatogenic microenvironment, this unique metabolic profile makes SCs a potential target for SAL toxicity. To date, no studies have explored whether SAL affects SCs, necessitating an evaluation of its potential toxicity or regulatory effects. This study focused on the impact of SAL on the mouse Sertoli cell line and its underlying mechanisms, aiming to characterize the cellular and metabolic responses of Sertoli cells to SAL at supraphysiological exposures, thereby providing a mechanistic reference for subsequent in vivo toxicokinetic and reproductive safety investigation.

## 2. Result

### 2.1. SAL Reduces Viability and Impairs the Expression of Function-Associated Genes of SCs

**Figure 1:**
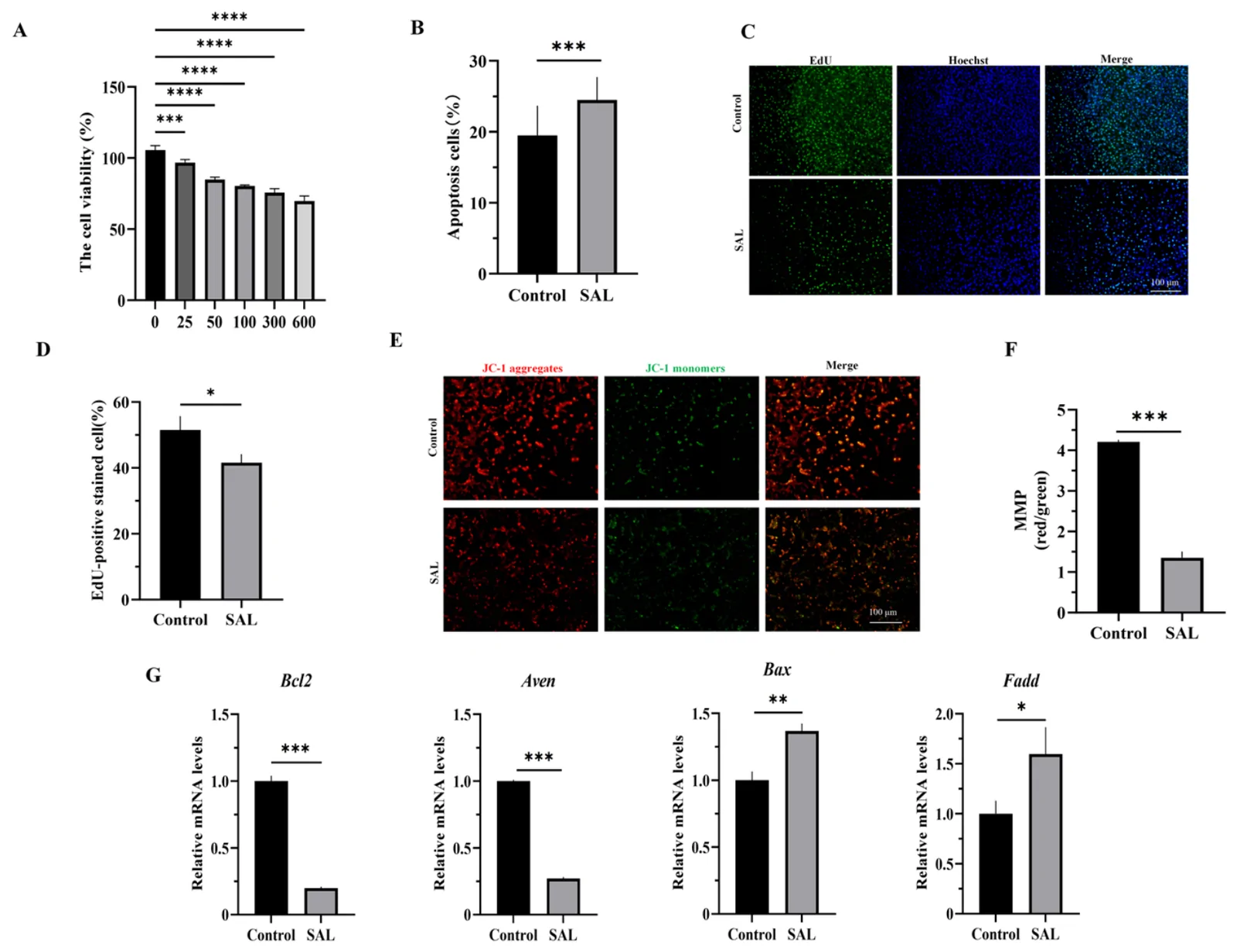
SAL inhibits proliferation and induces apoptosis in TM4 cells. (**A**) The CCK-8 assay was performed to detect the viability of TM4 cells treated with 0, 25, 50, 100, 300 and 600 μM SAL. (n = 3) (**B**) Quantitative analysis of the percentage of apoptotic cells in the Control and 600 μM SAL treatment groups (denoted as the SAL group in the following figures) by flow cytometry (n = 3). (**C**) Cell proliferation was detected by EdU staining in the Control and SAL groups (n = 3). Bar: 100 μm. (**D**) The proportion of EdU-positive stained cells in (**C**). (**E**) Mitochondrial membrane potential (MMP) was detected by the JC-1 fluorescent probe in the Control and SAL groups. JC1-monomers and JC1-aggregates produce green and red fluorescence, respectively. (n = 3). Bar: 50 μm. (**F**) MMP in (**E**) was quantified by measuring the ratio of fluorescence intensity of JC1-aggregates to JC1-monomers. (**G**) Relative mRNA levels of anti-apoptosis genes (*Bcl2* and *Aven*) and pro-apoptosis genes (*Bax* and *Fadd*) in Control and SAL groups (n = 3). For (**A**), one-way ANOVA (followed by Tukey’s post-hoc multiple comparison test) was used. For (**B**,**D**,**F**,**G**), Student’s unpaired *t*-test was used. All data are presented as mean ± SD. * *p* < 0.05, ** *p* < 0.01, *** *p* < 0.001, **** *p* < 0.0001 indicate statistical significance at different levels.

Preliminary experiments showed that the half-maximal Inhibitory Concentration (IC_50_) of SAL in TM4 cells was 848.9 µM (Figure S1). Given the poor cell viability observed at 800 µM, TM4 cells were treated with a series of SAL concentrations below 800 µM (0, 25, 50, 100, 300, and 600 µM), and cell viability was assessed by CCK-8 assay. The results indicated that, compared to the Control group, cell viability decreased by 8.29%, 19.60%, 23.82%, 28.26%, and 30.81% after treatment with 25 μM, 50 μM, 100 μM, 300 μM, and 600 μM SAL, respectively (Figure 1A) and the proportion of apoptotic cells increased by 25.36% (Figure 1B and Figure S2) in the 600 μM SAL treatment group (noted as SAL group below) compared with that of the Control group. EdU staining results demonstrated that, compared to the Control group, the SAL group exhibited a significant decrease in cell proliferation ability (Figure 1C,D). A decrease in mitochondrial membrane potential (MMP) is a hallmark of apoptosis. The results showed that compared with the Control group, the MMP of SCs decreased by 67.93% in the SAL group (Figure 1E,F). Furthermore, compared with the Control group, the SAL group showed increased expression of the pro-apoptotic genes *Bax* and *Fadd* by 36.79% and 59.77%, respectively, while the anti-apoptotic genes *Bcl-2* and *Aven* were downregulated by 80.06% and72.82%, respectively (Figure 1G). These findings collectively confirm that SAL induces apoptosis in TM4 cells.

**Figure 2:**
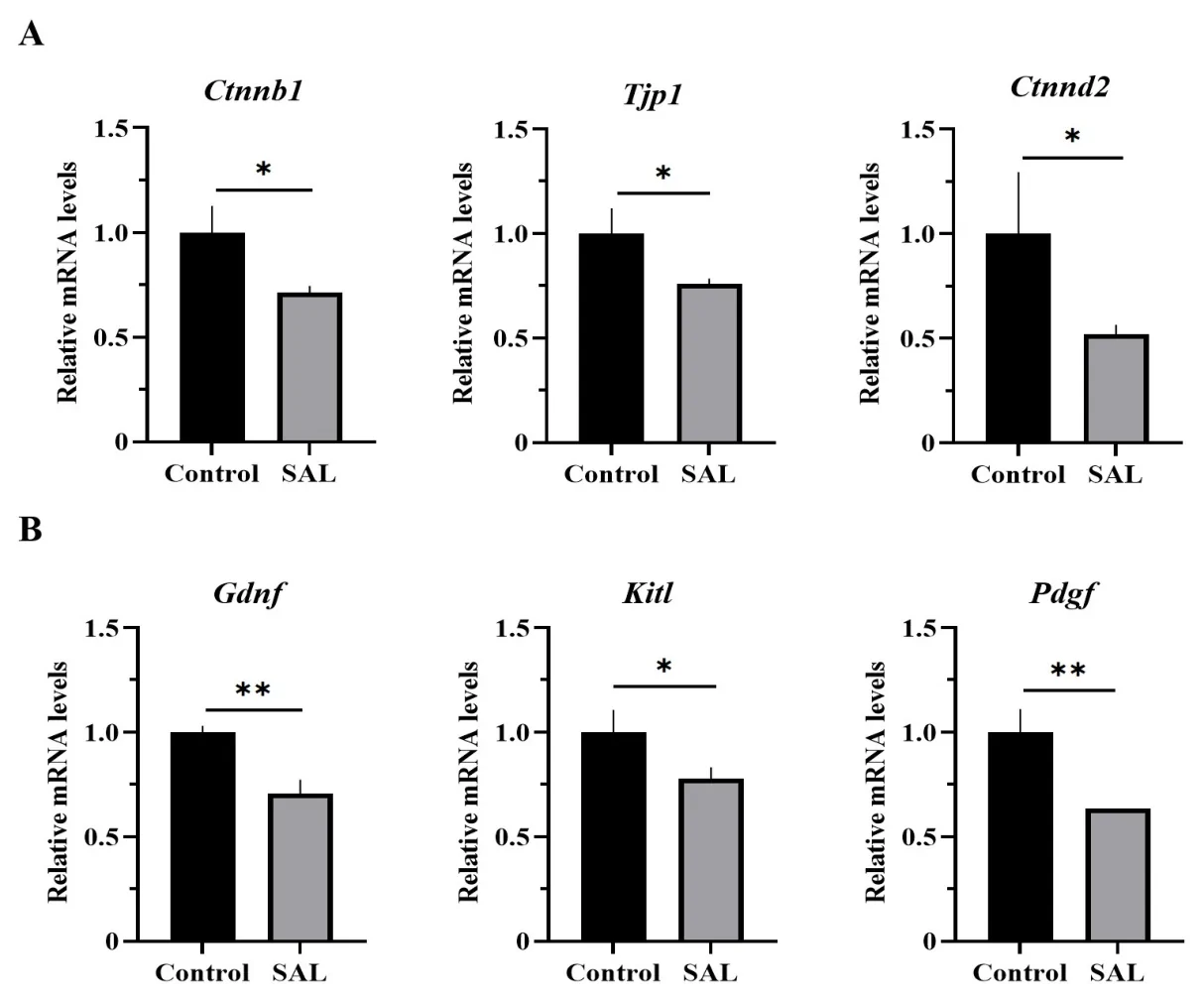
The impact of SAL treatment on the mRNA expression of Sertoli cell function-related genes in TM4 cells. (**A**) Relative mRNA levels of several Sertoli cell secretory factors responsible for spermatogenesis in Control and SAL groups (n = 3). (**B**) Relative mRNA levels of several genes encoding BTB-related proteins in Control and SAL groups (n = 3). Student’s unpaired *t*-test. Data presented are expressed as mean ± SEM from three independent experiments. * *p* < 0.05 and ** *p* < 0.01 indicate statistical significance at different levels.

Given that SCs support spermatogenesis through paracrine signaling and by forming the BTB, the mRNA expressions of several spermatogenesis-related factors secreted by SCs, including *Gdnf*, *Kitl*, and *Pdgf*, were detected. The results showed that compared with the Control group, the expression levels of *Gdnf*, *Kitl*, and *Pdgf* decreased by 36.68%, 22.26% and 29.41% in the SAL group, respectively (Figure 2A). Similarly, 600 μM SAL treatment repressed the expression of several BTB formation-related genes, including *Ctnnb1*, *Tjp1*, and *Ctnnd2* (Figure 2B), suggesting that SAL impairs the expression of both BTB formation-related and paracrine function-related genes.

### 2.2. Dynamic Transcriptome Changes in Mouse SCs Treated with SAL

**Figure 3:**
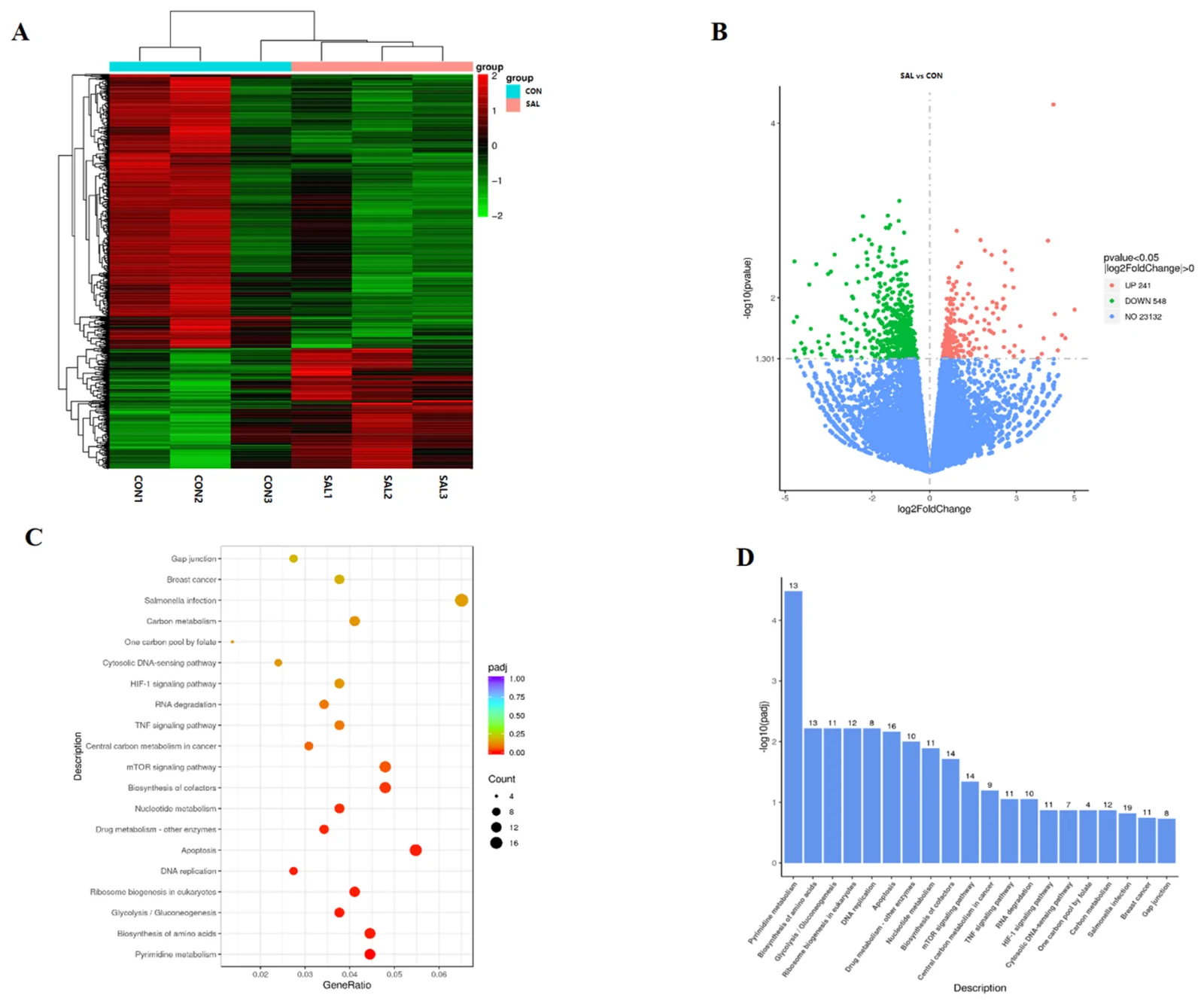
Transcriptome analysis of TM4 cell line between Control and SAL groups. (**A**) Heatmap of significantly differential genes (DEGs) clustering in Control and SAL groups; Con1–Con3 and SAL1–SAL3 represent three independent replicate samples for the Control and SAL groups, respectively; (**B**) volcano plot of DEGs in Control and SAL groups; (**C**) KEGG pathway enrichment scatter plot of DEGs; (**D**) bar plot of KEGG pathway enrichment for DEGs. The numbers above the bars indicate the number of DEGs enriched in the respective pathways.

To further explore the mechanism by which SAL affects the viability of TM4 cells, mRNA from the Control and SAL groups was collected and sequenced. The high Pearson correlations (R^2^ > 0.946) and clear PCA separation collectively demonstrate good sequencing quality and acceptable sample consistency for downstream analysis (Figure S3). A total of 789 differentially expressed transcripts were identified in the Control versus (vs.) SAL groups, including 548 downregulated genes and 241 upregulated genes. (Figure 3A,B, Table S2). The accuracy of the sequencing data was confirmed by real-time PCR (Figure S4), and then KEGG enrichment analysis was applied to identify the altered biological processes. The top 5 significantly enriched pathways included Pyrimidine metabolism (mmu00240), Biosynthesis of amino acids (mmu01230), Glycolysis/Gluconeogenesis (mmu00010), Ribosome biogenesis in eukaryotes (mmu03008), and DNA replication (mmu03030) (Figure 3C,D, Table S3).

### 2.3. SAL Treatment Impaired the Glycolytic Pathways in TM4 Cells

**Figure 4:**
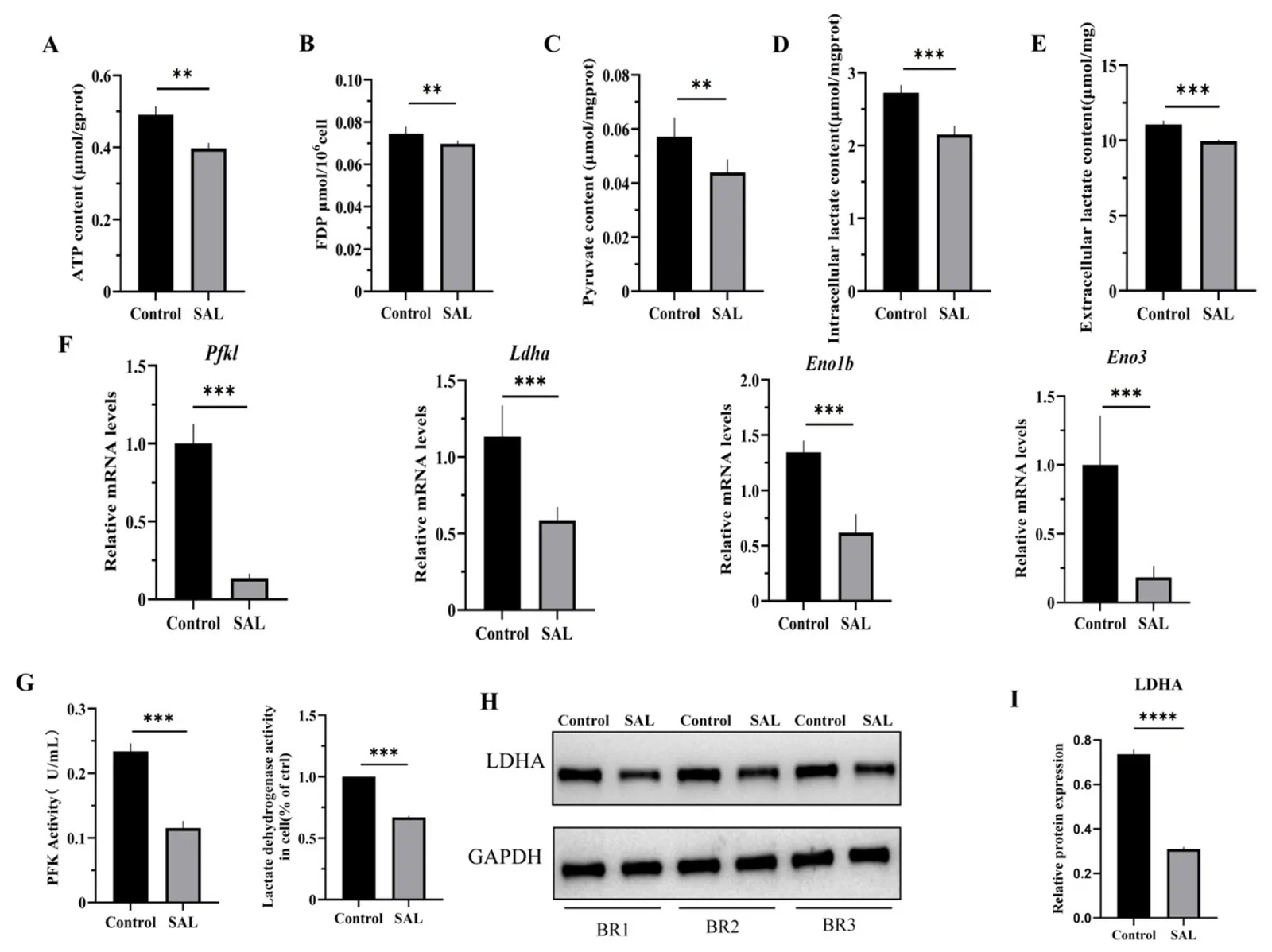
SAL treatment impaired the glycolytic pathways in TM4 cells. (**A**) ATP content in Control and SAL groups (n = 3). (**B**–**E**) FDP (**B**), pyruvate (**C**), intracellular (**D**) and extracellular (**E**) lactate contents in Control and SAL groups (n = 3). (**F**) Relative mRNA levels of four glycolysis pathway-related genes screened from RNA-seq using real-time PCR (n = 3). (**G**) The activities of PFK and LDH in Control and SAL groups, and the intracellular LDH enzymatic activity, were normalized to the Control group (n = 3). (**H**) Protein expression of LDHA. BR1, BR2 and BR3 represent three independent biological replicates, the same as below (n = 3). (**I**) Optical density analysis of the Western blot results shown in (**B**) (n = 3). Student’s unpaired *t*-test. Data are expressed as mean ± SEM from three independent experiments. ** *p* < 0.01, *** *p* < 0.001 and **** *p* < 0.0001 indicate statistical significance at different levels.

Glycolysis is the primary pathway for cells to obtain energy. KEGG pathway enrichment analysis revealed that the glycolysis pathway was significantly affected after SAL treatment (Figure 3C,D). To validate this finding, the levels of ATP and several important key glycolytic metabolites, including fructose-1,6-bisphosphate (FDP), pyruvate, and lactate, were measured in Control and SAL groups. The results showed that SAL treatment resulted in an 18.98% decrease in ATP levels compared to the Control group (Figure 4A). In addition, the contents of FDP, pyruvate, intracellular and extracellular lactate decreased by 6.34%, 23.02%, 21.12% and 10.06% in the SAL group compared with those of the Control group (Figure 4B–E). Four key genes (*Pfkl*, *Ldha*, *Eno3*, and *Eno1b*) in the glycolytic pathway were found to be significantly downregulated in the SAL group compared with those of the Control group from RNA-Seq (Figure S5). To further validate the expression levels of these genes, real-time PCR was used for their quantitative detection, and all these genes were repressed after SAL treatment (Figure 4F). The activities of PFK and LDH were also impaired (Figure 4G), which indicated that SAL disrupts the glycolysis process in TM4 cells. The protein expression levels of LDHA were assessed, revealing results similar to those of the mRNA expression. (Figure 4H,I and Figure S6A,B).

### 2.4. Overexpression of Ldha Partially Rescued Impaired Viability and Expression of Function-Associated Genes in SAL-Treated SCs

**Figure 5:**
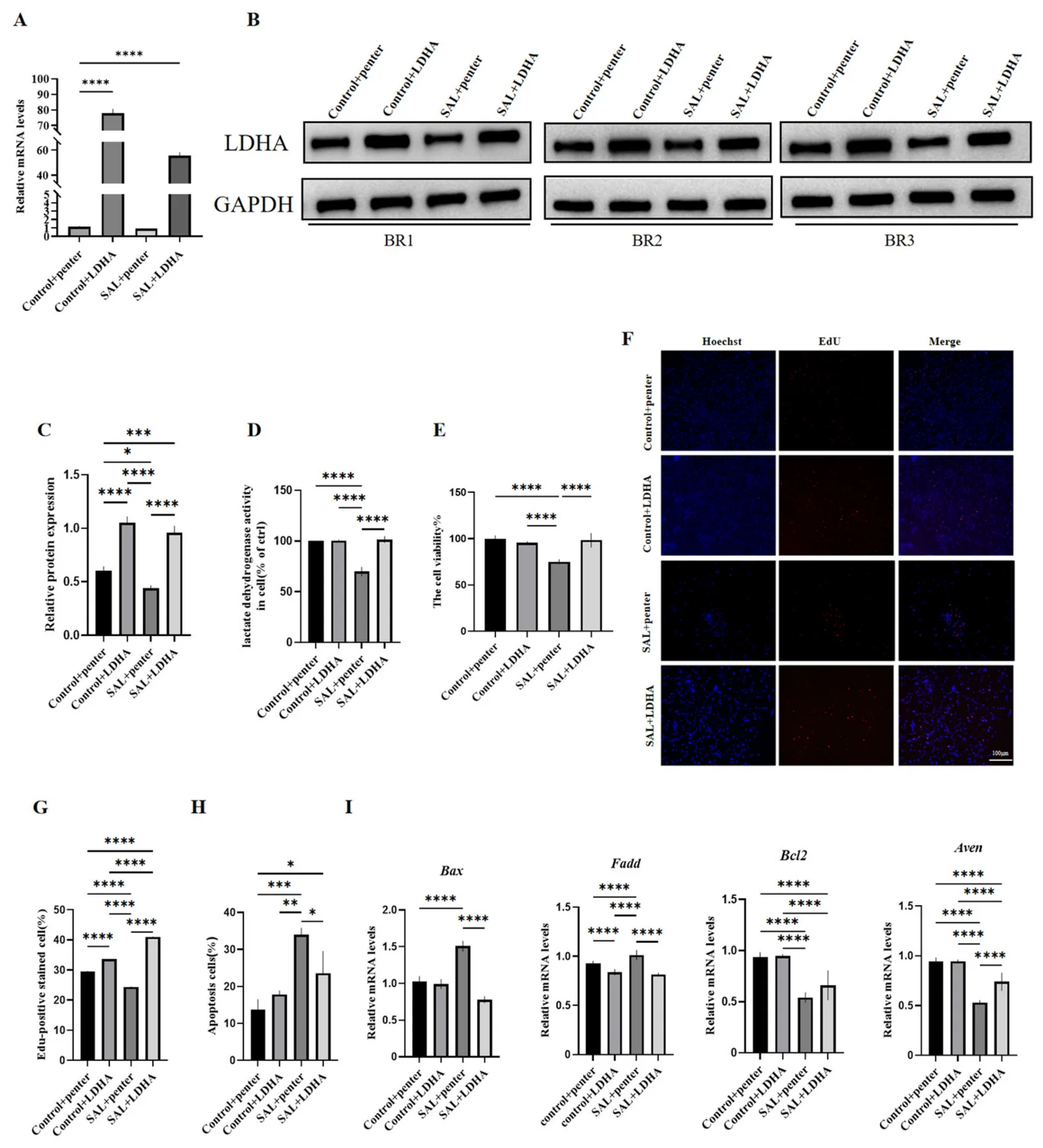
Overexpression of *Ldha* reversed SAL-induced cell viability suppression in TM4 cells. (**A**) Relative mRNA expression levels of *Ldha* (n = 3). (**B**) Relative protein levels in the different groups (n = 3). (**C**) Optical density analysis of the Western blot results shown in (**B**) (n = 3). (**D**) Intracellular LDH enzymatic activity normalized to the Control+penter group (n = 3). (**E**) Relative cell viability of SCs detected by CCK-8 assay (n = 3). (**F**,**G**) Cell proliferation was detected by EdU staining (n = 3). (**F**) The proportion of EdU-positive stained cells in (**G**). (**H**) Quantitative analysis of the percentage of apoptotic cells in the four groups by flow cytometry. n = 3. (**I**) Relative transcript abundance of apoptosis-related genes, including *Bax*, *Fadd*, *Aven*, and *Bcl-2* (n = 3). One-way ANOVA (followed by Tukey’s post-hoc multiple comparison test). Data are expressed as mean ± SEM from three independent experiments. * *p* < 0.05, ** *p* < 0.01, *** *p* < 0.001 and **** *p* < 0.0001 indicate statistical significance at different levels.

*Ldha* encodes LDHA, the core catalytic subunit that governs intracellular LDH enzymatic activity and lactate generation in SCs. To determine whether *Ldha* is the target gene responsible for SAL-mediated inhibition of SC viability, this gene was overexpressed in SAL-treated SCs. Briefly, cells were divided into four experimental groups: the 0.1%DMSO-treated group transfected with empty vector (Control+penter group), the DMSO-treated group overexpressing *Ldha* (Control+LDHA group), the 600 μM SAL-treated group transfected with empty vector (SAL+penter group), and the SAL-treated group overexpressing LDHA (SAL+LDHA group). The results showed that, compared with the SAL+penter group, both the mRNA and protein expression of *Ldha*, as well as the activity of LDH were significantly upregulated in the SAL+LDHA group (Figure 5A–D and Figure S6). Cell viability in the SAL+LDHA group significantly increased by 31.01% compared to that of the SAL+penter group (Figure 5E). EdU staining results demonstrated that compared to the SAL+penter group, the SAL+LDHA group exhibited a significant increase in cell proliferation ability (Figure 5F,G), and compared with the SAL+penter group, the proportion of apoptotic cells decreased by 30.69% in the SAL+LDHA group (Figure 5H and Figure S7). Furthermore, compared with the SAL+penter group, the expression of the pro-apoptotic genes *Bax* and *Fadd* was downregulated by 48.38% and 19.51% in the SAL+LDHA group, respectively, while the expression of the anti-apoptotic genes *Bcl-2* and *Aven* was upregulated by 21.97% and 39.32%, respectively (Figure 5I).

**Figure 6:**
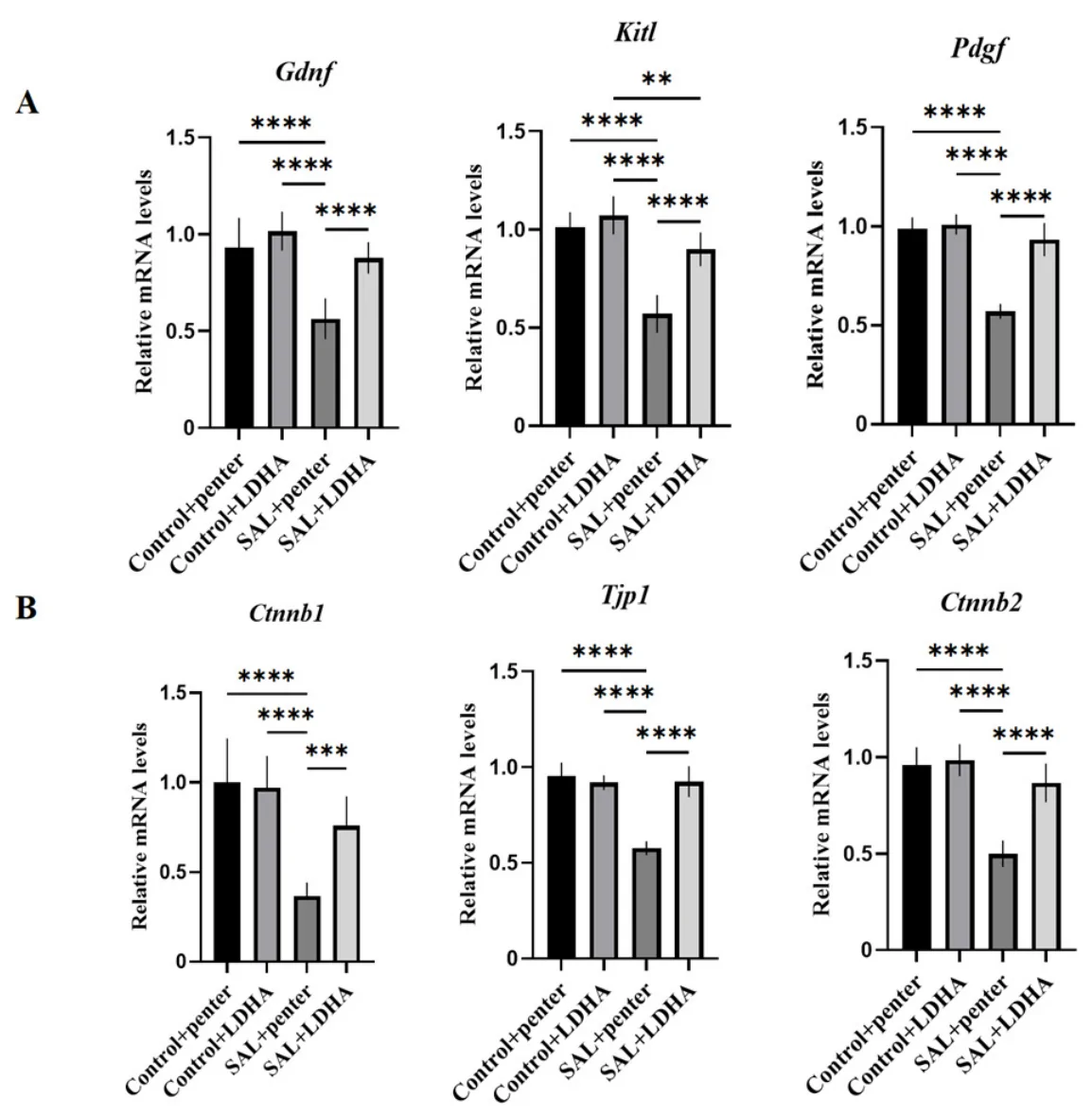
Overexpression of *Ldha* reversed the expression of several Sertoli cell function-related genes. (**A**) Relative mRNA levels of spermatogenesis-supporting secreted factors *Gdnf*, *Kitl*, and *Pdgf* (n = 3). (**B**) Relative mRNA expression of blood–testis barrier (BTB) formation-associated genes *Ctnnb1*, *Tjp1*, and *Ctnnd2* (n = 3). One-way ANOVA (followed by Tukey’s post-hoc multiple comparison test). Data are expressed as mean ± SEM from three independent experiments. ** *p* < 0.01, *** *p* < 0.001 and **** *p* < 0.0001 indicate statistical significance at different levels.

In addition, compared with the SAL+penter group, the expression of several Sertoli cell function-related genes, including *Gdnf*, *Kitl*, *Pdgf*, *Ctnnb1*, *Tjp1* and *Ctnnb2*, increased by 36.68%, 22.26%, 29.41%, 106.90%, 60.18%, and 73.68% in the SAL+LDHA group, respectively (Figure 6A,B). These findings collectively substantiate that overexpressing *Ldha* partially rescued the compromised viability and impaired expression of Sertoli cell function-related genes in TM4 cells.

### 2.5. Overexpression of Ldha Partially Rescued the Impaired Production of ATP and Lactic Acid in SAL-Treated SCs

**Figure 7:**
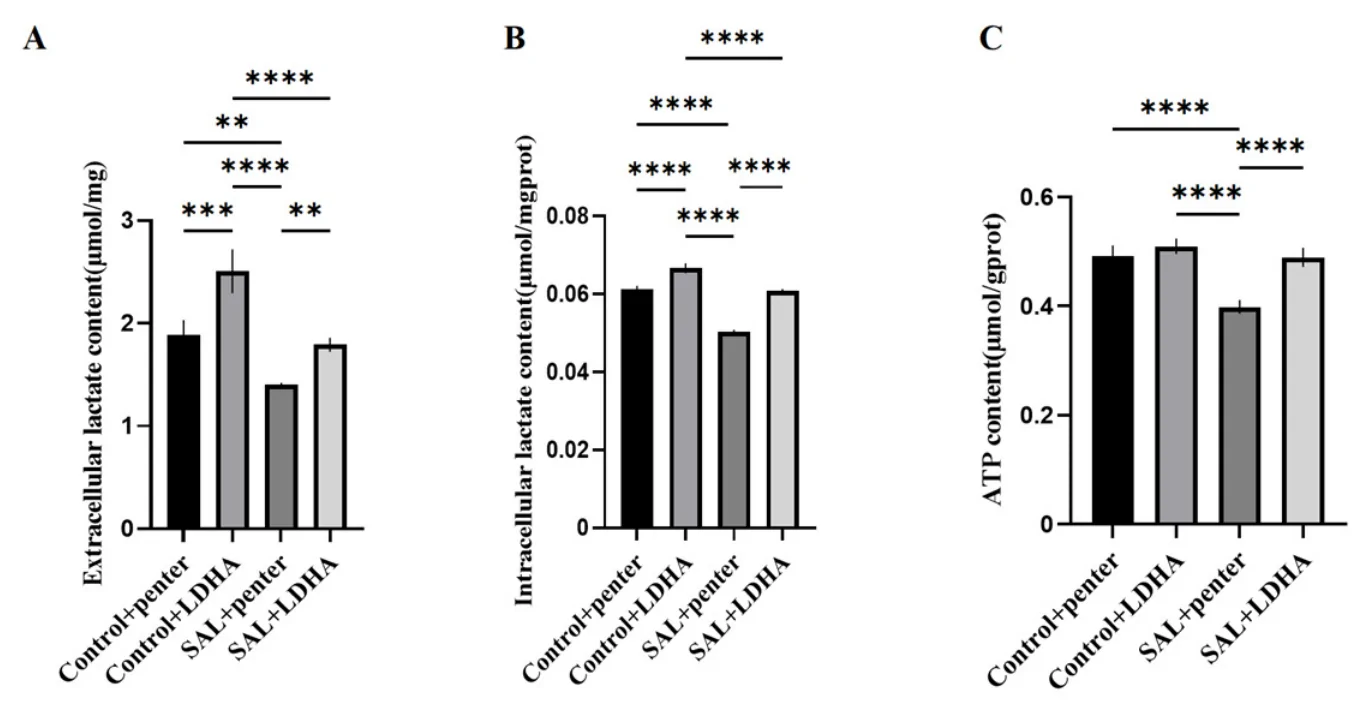
Overexpression of *Ldha* partially rescued impaired production of ATP and lactic acid in SAL-treated TM4 cells. (**A**) Extracellular lactic acid levels (n = 3). (**B**) Intracellular lactic acid levels (n = 3). (**C**) ATP content of SCs (n = 3); one-way ANOVA followed by Tukey’s post-hoc multiple comparison test. Data are expressed as mean ± SEM from three independent experiments. ** *p* < 0.01, *** *p* < 0.001 and **** *p* < 0.0001 indicated statistical significance at different levels.

To verify whether overexpressing *Ldha* could partially restore SAL-impaired glycolytic metabolism, we detected glycolytic metabolites and glycolysis-related gene expression in SAL-treated SCs. The results showed that, compared with the SAL+penter group, extracellular and intracellular lactic acid levels were significantly increased by 27.75% and 20.73% in the SAL+LDHA group (Figure 7A,B). In addition, overexpressing *Ldha* markedly restored ATP content by 22.85% in the SAL+LDHA group (Figure 7C).

## 3. Discussion

As the principal bioactive constituent of *Rhodiola rosea*, SAL has been established to exert a dual regulatory effect on cell viability. It has also been shown to alleviate cellular damage induced by adverse factors such as hypoxia, excessive oxidative stress, and PM2.5 exposure [22,23,24]. Conversely, in specific cell types, especially tumor cells, SAL inhibits proliferation while promoting apoptosis [25,26,27]. However, to date, little is known about the effects of SAL on cell types associated with reproductive function under non-stress conditions. This study investigated the regulatory effects and underlying mechanisms of SAL on mouse SCs, providing foundational data for further research on the role of SAL in reproduction.

Over the years, numerous studies have demonstrated that SAL inhibits tumor cell viability by suppressing proliferation and promoting apoptosis, and that this inhibitory effect is positively correlated with SAL concentration in various tumor cells, such as HT29 colorectal cells (0–200 μM SAL) [28], chronic myeloid leukemia cells (0–100 μM), and human lung cancer cells (0–8000 μM) [29]. The present study showed that within the concentration range of 0–600 μM, cell viability decreased significantly with increasing SAL concentrations, a trend highly consistent with the tumor-selective inhibitory properties reported in the literature. Compared with the Control group, the SAL group exhibited suppressed proliferation and increased apoptosis at both the cellular and molecular levels, suggesting that the inhibitory effect of SAL on TM4 cell viability is achieved through inhibiting cell proliferation and promoting apoptosis.

Paracrine secretion and participation in the formation of the blood–testis barrier are now understood to be two important ways in which SCs regulate spermatogenesis. Studies have reported that under stress conditions, SAL can promote the paracrine function of skeletal muscle cells and mesenchymal stem cells [30,31]. In addition, SAL has been shown to repair the damaged blood–testis barrier in streptozotocin-induced type 1 diabetic male mice [32]. However, the results of the present study indicated that multiple genes encoding paracrine factors and blood–testis barrier components in SCs were significantly downregulated following SAL treatment. Indeed, whether this finding suggests that SAL inhibits the paracrine function and blood–testis barrier integrity of SCs under non-stressed conditions remains to be further verified experimentally.

Research has shown that, unlike most somatic cells, SCs predominantly rely on aerobic glycolysis for their metabolism. Lactate produced through glycolysis not only serves as the primary energy source for SCs themselves but also plays a critical role in regulating the proliferation, differentiation, and anti-apoptotic processes of spermatogenic cells. In glycolysis, LDH catalyzes the final conversion of pyruvate to lactate, thus playing a key role throughout the glycolytic pathway [9,10]. *Ldha* encodes LDHA, the key subunit of LDH. Moreover, studies have demonstrated that *Ldha* deficiency inhibits Sertoli cell proliferation and promotes their apoptosis [10]. Mice with Sertoli cell-specific deletion of *Ldha* exhibit significantly reduced fertility and impaired spermatogenesis [10,33]. In the present study, transcriptome sequencing results showed that, compared with the control group, *Ldha* expression in SCs was significantly downregulated following treatment with 600 μmol/L SAL. Further analysis of the glycolytic pathway revealed that LDHA protein expression, LDH enzymatic activity, and intracellular and extracellular levels of lactate and ATP were all decreased. Moreover, overexpression of *Ldha* in SAL-treated SCs promoted cell proliferation, inhibited apoptosis, restored cell viability, and increased intracellular and extracellular lactate and ATP levels. These findings collectively indicate that the inhibitory effect of SAL on TM4 cell viability is largely attributable to the LDHA-regulated glycolytic pathway.

In addition to *Ldha*, several other genes in the glycolytic pathway, including *Eno1b*, *Eno3*, and *Pfkl*, were significantly downregulated in the SAL-treated group. These genes all encode core enzymes of the glycolytic pathway: *Pfkl* encodes phosphofructokinase-1 (PFK-1), which catalyzes the conversion of fructose-6-phosphate to fructose-1,6-bisphosphate, representing the rate-limiting regulatory step of glycolysis [16,21]; both *Eno1b* and *Eno3* encode enolase, which together catalyze the dehydration of 2-phosphoglycerate to generate the high-energy substrate phosphoenolpyruvate, laying the foundation for subsequent substrate-level phosphorylation to produce ATP [34,35]. Importantly, our study showed that following SAL treatment, the levels of fructose-1,6-bisphosphate and pyruvate, two key glycolytic metabolites generated by the enzymatic reactions catalyzed by the above-mentioned gene products, were also significantly decreased. This finding suggests that the inhibitory effect of SAL on TM4 cell viability may be associated with the global suppression of the glycolytic pathway. However, whether this inhibitory effect is specifically attributable to one or several of these genes requires further experimental validation. Notably, cancer cells share a similar aerobic glycolytic metabolic profile with SCs. SAL has been found to suppress cell viability by inhibiting glycolysis in various cancer cell types [20,21]. Taken together with the findings of the present study, these observations suggest that SAL may possess broad-spectrum metabolic regulatory activity targeting aerobic glycolysis.

In addition to glycolysis, transcriptome sequencing results indicated that SAL treatment broadly affected other key metabolic processes in SCs, such as nucleotide metabolism and amino acid biosynthesis. Moreover, several core biological events related to cell fate, including DNA replication, ribosome biogenesis in eukaryotes, and RNA degradation, as well as multiple important signaling pathways, such as the mTOR signaling pathway, HIF-1 signaling pathway, and TNF signaling pathway, were all significantly impacted, which suggests that SAL may influence Sertoli cell fate through multiple pathways and mechanisms. In addition, the mTOR and HIF-1 signaling pathways have been reported to regulate LDHA expression [36,37], and whether the inhibitory effect of SAL on LDHA is associated with these pathways requires further experimental validation.

Although this in vitro study demonstrates that supraphysiological concentrations of SAL, particularly the 600 μM exposure condition impair TM4 Sertoli cell viability, no pharmacokinetic or tissue-exposure data are currently available to indicate that free salidroside concentrations approaching 600 μM can be attained in murine or human testicular tissue or systemic plasma under realistic oral/intravenous dosing regimens. Given the acknowledged disparity between in vitro exposure levels and likely in vivo free-drug exposure, the present findings should not be construed as direct evidence of reproductive hazard at therapeutic doses. Rather, they establish a mechanistic hypothesis that warrants validation through dedicated in vivo pharmacokinetic profiling and reproductive toxicity assessments conducted at exposure levels aligned with physiologically achievable drug concentrations.

## 4. Materials and Methods

### 4.1. Cell Culture and Treatment

The mouse testicular Sertoli cell line (TM4 cells) was purchased from Procell Life Science Technology Co., Ltd. (CL-0456, Wuhan, China). Cells were cultured using DMEM/F12 medium (11320033, Gibco, New York, NY, USA) containing 10% fetal bovine serum (10099141, Gibco, New York, NY, USA) with 1% penicillin–streptomycin solution (15140122, Thermo Fisher Scientific, Pittsburgh, PA, USA). SAL (S420418, Aladdin, Shanghai, China) was dissolved in DMSO (D8418, Sigma-Aldrich, St. Louis, MO, USA) to prepare a 60 mM stock solution, which was subsequently diluted in culture medium to achieve the final working concentrations (25, 50, 100, 300, and 600 µM). The final DMSO concentration was uniformly maintained at 0.1% (*v*/*v*) across all treatment groups. Cells treated with 0.1% DMSO were designated as the Control group. Given the exploratory dose–response scope of this experiment, no pharmacological positive control was included; the untreated group served as the baseline reference for all concentration comparisons. All cells were maintained at 37 °C in a humidified atmosphere with 5% CO_2_ for 48 h prior to further experimental procedures.

### 4.2. Cell Counting Kit-8 (CCK-8) Assay

Cells treated with 0, 25, 50, 100, 300 and 600 μM SAL were seeded into 96-well plates at a density of approximately 4 × 10^4^ cells per well. Cell viability was then assessed using a Cell Counting Kit-8 (CCK-8) Kit (Med Chem Express, HY-K0301, Shanghai, China) according to the manufacturer’s instructions. The CCK-8 assay employs a water-soluble tetrazolium salt, WST-8 (2-(2-methoxy-4-nitrophenyl)-3-(4-nitrophenyl)-5-(2,4-disulfophenyl)-2H-tetrazolium), to evaluate cell viability. The optical density at 450 nm was measured using a spectrophotometer (Thermo Scientific™, model 1530-00183, Waltham, MA, USA). Cell viability was calculated as specified by the manufacturer. Each treatment group consisted of three independent biological replicates, and each biological replicate included three technical replicates.

### 4.3. 5-Ethynyl-2′-deoxyuridine (EdU) Staining Assay

EdU staining of TM4 cells of the Control and 600 μM SAL-treated groups was performed using the BeyoClick™ EdU Cell Proliferation Kit with Alexa Fluor 488 (Beyotime, C0071S, Shanghai, China), as per the manufacturer’s protocol. Cells were incubated with 10 µM EdU solution for 2 h. Following incubation, cells were fixed with 4% paraformaldehyde (P0099, Beyotime Biotechnology, Shanghai, China) and permeabilized with 0.3% Triton X-100 (P0096, Beyotime Biotechnology, Shanghai, China) at room temperature for 15 min. After washing three times with wash buffer PBS containing 3% BSA (P0651, Beyotime Biotechnology, Shanghai, China), the click reaction solution (composed of Click Reaction Buffer Alexa Fluor 488 azide, CuSO_4_, and click additive solution) was added, and cells were incubated in the dark for 30 min. Nuclei were then counterstained with Hoechst (Solarbio, C0031, Beijing, China). Images were acquired using a ZEISS microscope (Zeiss, LSM800, Iena, Germany). Each treatment group consisted of three independent biological replicates, and each biological replicate included three technical replicates.

### 4.4. Flow Cytometry Assay

Control and 600 μM SAL-treated TM4 cells were seeded in 6-well plates at a density of 2 × 10^5^ cells/mL. At 48 h post-inoculation, the cells were digested with EDTA-free trypsin (BL526A, Biosharp, Beijing, China) for 30 s, and the digestion was terminated by adding complete culture medium. The cell suspension was collected and centrifuged at 250× *g* for 5 min. After removal of the supernatant, the cell pellets were washed twice with phosphate-buffered saline (PBS), with centrifugation at 250× *g* for 5 min after each wash. According to the manufacturer’s instructions for the Annexin V-APC/PI Apoptosis Detection Kit (KGA1107, KeyGEN BioTECH, Nanjing, China), the cell pellets were resuspended in 500 μL of Binding Buffer, followed by sequential addition of 5 μL of Annexin V-APC and 5 μL of propidium iodide (PI). The mixture was gently vortexed and incubated at room temperature for 10 min in the dark. Finally, apoptosis was analyzed using a flow cytometer, and the data were processed with CytExpert software (version 2.3). Each treatment group consisted of three independent biological replicates, and each biological replicate included three technical replicates.

### 4.5. Mitochondrial Membrane Potential (MMP) Detection

Control and 600 μM SAL-treated TM4 cells were seeded onto polylysine-coated slides in 24-well plates. MMP was assessed using a Mitochondrial Membrane Potential Assay Kit (C2006, Beyotime, Shanghai, China) according to the manufacturer’s instructions. Briefly, cells were incubated with the fluorescent probe JC-1 (5,5′,6,6′-Tetrachloro-1,1′,3,3′-tetraethyl-imidacarbocyanine iodide) at 37 °C for 20 min. Following incubation, the supernatant was removed, and the slides were washed twice with diluted 1 × JC-1 staining buffer. Fluorescence was visualized using a fluorescence microscope (Zeiss, Axio Vert.A1, Iena, Germany), with JC-1 aggregates detected at excitation/emission wavelengths of 520 nm/595 nm (red) and monomers at 490 nm/530 nm (green). Fluorescence intensity was quantified using a microplate reader (SpectraMax iD5, Molecular Devices, Shanghai, China). MMP was evaluated as the ratio of red fluorescence intensity (JC-1 aggregates) to green fluorescence intensity (JC-1 monomers). Each treatment group consisted of three independent biological replicates, and each biological replicate included three technical replicates.

### 4.6. RNA Extraction, cDNA Synthesis, and Real-Time PCR

Total RNA was extracted from Control and 600 μM SAL-treated TM4 cells (5 × 10^5^ to 1 × 10^6^ cells per sample) using TRIzol reagent (Invitrogen, Carlsbad, CA, USA) and reverse transcribed into cDNA using PrimeScriptTM RT Reagent Kit (R1223, Nanjing Vazyme Biotech Co., Ltd., Nanjing, China) following the manufacturer’s protocol. Primer design was completed online using Pick Primers (version: 10.4.0) from NCBI, and the sequences are detailed in Table S1. Real-time PCR was performed using the ChamQ Universal SYBR qPCR Master Mix kit (Q711-02, Nanjing Vazyme Biotech Co., Ltd., Nanjing, China) with a reaction system of 20 μL, which includes 1 μL of cDNA, 0.5 μL each of forward and reverse primers, 10 μL of 2 × ChamQ Universal SYBR qPCR Master Mix, and 8 μL of ddH_2_O. *Gapdh* was used as the reference gene, and the fold change in gene expression was quantified using the comparative C_T_ method. Each treatment group consisted of three independent biological replicates, and each biological replicate included three technical replicates.

### 4.7. Transcriptome Sequencing and Data Analysis

Total RNA from Control and 600 μM SAL-treated TM4 cells (n = 3 for each group) was collected and RNA integrity was confirmed on an Agilent 2100 Bioanalyzer (Agilent Technologies, Inc., Santa Clara, CA, USA) (RIN ≥ 8.0; 28S/18S ≥ 1.8). The cDNA libraries were sequenced on the Illumina NovaSeq series sequencing platform. Following library construction, each library was first quantified using a Qubit 2.0 Fluorometer (Thermo Fisher Scientific, Waltham, MA, USA) and diluted to 1.5 ng/µL. Insert size was then assessed on an Agilent 2100 Bioanalyzer. Libraries with an effective concentration > 1.5 nM were accepted for downstream sequencing to ensure library quality. A total of 3.87–5.04 × 10^7^ clean reads per sample were obtained after fastp filtering (Q20 ≥ 97.9%, Q30 ≥ 96.1%). Reads were aligned to the mouse reference GRCm38 (Ensembl M19) with HISAT2 (version: 2.0.5); uniquely mapped fractions ranged from 88.4–89.8%. Gene-level counts were obtained with featureCounts (version: 1.50-p3). Differential expression was performed in DESeq2 (version: 1.20.0) using median-of-ratios normalization and a negative-binomial model with default dispersion shrinkage. DEGs were defined as padj < 0.05 (Benjamini–Hochberg) and |log_2_ fold change| ≥ 1.0. PCA, Pearson correlation, and hierarchical clustering were performed on the log_2_(FPKM + 1)-transformed expression matrix using R (version 3.5.0) and visualized with the ggplot2 and pheatmap packages. Kyoto Encyclopedia of Genes and Genomes (KEGG) pathway enrichment analysis of these differentially expressed genes was performed using the clusterProfiler R package. KEGG terms with an adjusted *p*-value < 0.05 were considered significantly enriched. The processed RNA-seq data are available in Tables S2 and S3, and the raw sequence reads have been deposited in the NCBI Sequence Read Archive (SRA) database.

### 4.8. Determination of Fructose-1,6-diphosphate (FDP), Pyruvate, and Lactic Acid Levels

The cell-containing medium (5 × 10^6^ cells/mL) of Control and 600 μM SAL-treated TM4 cells was centrifuged at 1000 rpm for 10 min. After discarding the supernatant, the cell pellet was washed twice with PBS, followed by centrifugation at 1000 rpm for 10 min. Cells were then resuspended in PBS and lysed using an ultrasonic cell crusher (JY92-IIN, SCIENTZ, Ningbo, China) in an ice-water bath. The determination of FDP, pyruvate, and lactate content was performed according to the instructions of a Fructose-1,6-bisphosphate (FDP) Content Assay Kit (BC2245, Solarbio, Shanghai, China), a Pyruvic Acid (PA) Content Assay Kit (BC2205, Solarbio, Shanghai, China) and a L-Lactic Acid (L-LA) Content Assay Kit (BC2235, Solarbio, Shanghai, China), respectively. Each treatment group consisted of three independent biological replicates, and each biological replicate included three technical replicates.

### 4.9. Measurement of Phosphofructokinase (PFK) and Lactate Dehydrogenase (LDH) Activity

The cell-containing medium (10^4^ cells/mL) from Control and 600 μM SAL-treated TM4 cells was centrifuged at 4000 rpm for 10 min. The cell pellet was harvested and resuspended in 150–200 μL of ice-cold PBS. Cell lysis was then performed using an ultrasonic homogenizer in an ice-water bath. Following this step, the cell lysates were centrifuged at 4 °C, 8000 rpm for 10 min. The supernatant was collected and kept on ice. PFK and LDH activities were measured using the PFK activity assay kit (BC0535, Solarbio, Shanghai, China) and LDH activity assay kit (BC0685, Solarbio, Shanghai, China), respectively, following the manufacturer’s instructions. Each treatment group consisted of three independent biological replicates, and each biological replicate included three technical replicates.

### 4.10. Measurement of ATP

Control and 600 μM SAL-treated TM4 cells were seeded into 6-well plates at a density of approximately 2 × 10^6^ cells per well and cultured for 48 h. The medium was then discarded and the cells were rinsed twice with pre-cooled PBS to remove residual medium. Subsequently, cells were digested with 0.25% trypsin-EDTA, and the digestion reaction was terminated with complete medium and the cells were harvested by centrifugation at 1000 rpm for 10 min. After discarding the supernatant, the cell pellet was washed twice with PBS. The washed cells were resuspended in 300–500 μL of ice-cold double-distilled water and lysed using an ultrasonic cell crusher (JY92-IIN, SCIENTZ, Ningbo, China) in an ice-water bath. The resulting lysate was heated in a boiling water bath for 10 min, vortexed for 1 min, and then centrifuged. ATP content in the supernatant was measured using an ATP Assay Kit (A095, Nanjing Jiancheng Bioengineering Institute, Nanjing, China) according to the manufacturer’s instructions. Each treatment group consisted of three independent biological replicates, and each biological replicate included three technical replicates.

### 4.11. Western Blot Analysis

The Sertoli cells were inoculated into 6-well cell plates with approximately 2 × 10^6^ cells/well. Subsequently, the cells were lysed using a buffer 100 mM Tris/HCl (RDD009, Sigma, St. Louis, MO, USA) pH 7.4, 3% SDS (15525017, Thermo Fisher Scientific, Pittsburgh, PA, USA), 10 mM DTT (R0861, Thermo Fisher Scientific, Pittsburgh, PA, USA), 17.3% glycerin (15514-011, Thermo Fisher Scientific, Pittsburgh, PA, USA), and 0.15% bromophenol blue (B5525, Sigma, St. Louis, MO, USA), supplemented with cOmpleteTM Protease Inhibitor Cocktail (04693116001, Roche, Basel, Switzerland). The protein concentration was quantified using a BCA Protein Assay Kit (Oriscience, PD101, Chengdu, China). Protein samples were mixed with loading buffer (Biosharp, BL502B, Beijing, China) and denatured by heating at 100 °C for 10 min in a metal bath. Equal amounts of protein (20 μg per lane) were loaded onto a 12% SDS-polyacrylamide gel for electrophoretic separation. Subsequently, the separated proteins were transferred onto a polyvinylidene difluoride (PVDF) membrane (Thermo Scientific, 88518, Waltham, MA, USA). The membrane was then blocked with 5% skim milk for 2 h at room temperature. Prior to antibody incubation, the PVDF membrane was cut into appropriate strips based on the molecular weight markers. Immunoblotting was performed using the following primary antibodies: anti-LDHA (1:1000, 19987-1-AP, Proteintech, Chicago, IL, USA), and anti-GAPDH (1:2000, 60004-1-Ig, Proteintech, Chicago, IL, USA) as an internal control. Protein bands were visualized and their intensities were quantified using TANON GIS software (V3.0, Tanon, Shanghai, China).

### 4.12. Ldha Overexpression Vector Construction and Plasmid Transfection

To construct the *Ldha* overexpression vector, the cloning vector pcDNA3.1(+) underwent double digestion with BamHI and EcoRI. The linearized vector and the mouse *Ldha* coding sequence (CDS) were then recombined using a gene recombination kit (C115-01, Vazyme, Nanjing, China). Finally, both the empty vector (pcDNA3.1(+)) and the recombinant plasmid (pcDNA3.1(+)-Ldha) were transfected into cells. When the cell confluence reached 60–70%, liquid A (125 μL Opti-DMEM (31985062, Thermo Fisher Scientific, Pittsburgh, PA, USA) + 3.75 μL lipo 3000 (L3000001, Thermo Fisher Scientific, Pittsburgh, PA, USA) co-incubated for 5 min) and liquid B (125 μL Opti-DMEM + 5 μL P3000 co-incubated for 5 min) were added to the 6-well plate. Cells from different experimental groups were cultured under identical conditions, including passage intervals and confluence levels. After 48 h, both cells and culture supernatants were collected for performing the assays described in Section 4.2, Section 4.3, Section 4.4, Section 4.5, Section 4.6, Section 4.7, Section 4.8, Section 4.9, Section 4.10 and Section 4.11, with the exception of transcriptome sequencing (Section 4.7).

### 4.13. Statistical Analysis

The results were analyzed using Student’s unpaired *t*-test or one-way analysis of variance (ANOVA) with Tukey’s multiple comparison test after verification of normality and homogeneity of variance. All data were expressed as mean ± standard error (mean ± SEM). * *p* < 0.05, ** *p* < 0.01, *** *p* < 0.001, **** *p* < 0.0001 indicated statistical significance at different levels.

## 5. Conclusions

This study provides hitherto undocumented evidence that salidroside regulates the viability of TM4 cells, identifying *Ldha* as a potential target gene responsible for SAL-mediated inhibition. These findings establish a foundation for further exploration of its toxicological implications and for the assessment of its potential risks in the reproductive field.

## Data Availability

The raw data of RNA-Seq have been deposited in the NCBI Sequence Read Archive (SRA) database with the accession number PRJNA1503457. Other data presented in this study are included in the published article and Supplementary Materials. Further inquiries can be directed to the corresponding author.

## Supplementary Materials

The following supporting information can be downloaded at https://www.mdpi.com/article/10.3390/molecules31193575/s1. Figure S1. Cell survival curves of TM4 cells treated with different concentrations of SAL (n = 3). *** *p* < 0.001 and **** *p* < 0.0001 indicate statistical significance at different levels. Figure S2. Apoptotic cells detected by flow cytometry in the Control and 600 μM SAL treatment groups (n = 3). The quantitative statistical analysis of apoptotic cell proportion from this panel is presented in Figure 1B. Figure S3. (A) Pearson correlation heatmap of the Control (A1–A3) and 600 μM SAL treatment (B6–B8) groups. (B) PCA score plot of the same samples. Figure S4. Transcriptome data reliability verification. Four genes with different expression tendencies were randomly selected from the RNA-seq data (A), and the expression levels were analyzed using real-time PCR (B) (n = 3). Student’s unpaired *t*-test. Data presented are expressed as mean ± SEM from three independent experiments. * *p* < 0.05, ** *p* < 0.01 and *** *p* < 0.001 indicated statistical significance at different levels. Figure S5. Expressive abundances of four glycolysis-related genes in RNA-seq data of Control vs. SAL group (n = 3). Student’s unpaired *t*-test. Data presented are expressed as mean ± SEM from three independent experiments. * *p* < 0.05, ** *p* < 0.01 and *** *p* < 0.001 indicated statistical significance at different levels. Figure S6. The original Western Blot images. Lanes 1–6 of A and B correspond to the original image of Figure 4H. Lanes 7–10 of panels A and B, together with panels C and D, correspond to the original image of Figure 5B. BR1, BR2 and BR3 represents three independent biological replicates, the same as below. Figure S7. Apoptotic cells were detected by flow cytometry in the four groups (n = 3). The quantitative statistical analysis of apoptotic cell proportion from this panel is presented in Figure 5F. Table S1. Sequences of real-time PCR primers. Table S2. Details of DEGs in Control vs. SAL group. Table S3. DEGs significantly enriched pathways by KEGG analysis in Control vs. SAL group.

## Abbreviations

The following abbreviations are used in this manuscript:

SAL	Salidroside
Ldha	Lactate dehydrogenase A
SCs	Sertoli cells
BTB	Blood–testis barrier
PDGF	Platelet-derived growth factor
GDNF	Glial cell line-derived neurotrophic factor
NF-κB	Nuclear factor kappa-B
PKM2	Pyruvate kinase isoenzyme M2
ENO1	Enolase 1
GLUT1	Glucose transporter 1
TM4 cells	The mouse testicular Sertoli cell line
CCK-8	Cell Counting Kit-8
EdU	5-Ethynyl-2′-deoxyuridine
MMP	Mitochondrial membrane potential
DEGs	Differentially expressed genes
KEGG	Kyoto Encyclopedia of Genes and Genomes
SRA	Sequence Read Archive
FDP	Determination of fructose-1,6-diphosphate
PFK	Phosphofructokinase
LDH	Lactate dehydrogenase
BCL2	B-cell lymphoma-2
BAX	BCL2-associated X, apoptosis regulator
ATP	Adenosine triphosphate
Fadd	Fas (TNFRSF6)-associated via death domain
Aven	Apoptosis, caspase activation inhibitor
Kitl	Kit ligand
Ctnnb1	Catenin (cadherin-associated protein), beta 1
Tjp1	Tight junction protein 1
Ctnnd2	Catenin (cadherin-associated protein), delta 2
