# Supplementary material for: Salidroside Represses the Viability of Mouse Sertoli Cell Line (TM4 Cells) via Repression of Ldha-Dependent Glycolysis
Source: Molecules. 2026 Oct 8;31(19):3575. doi: 10.3390/molecules31193575 (PMC13666587; doi:10.3390/molecules31193575)
Supplement: Supplementary file 1 [file molecules-31-03575-s001.zip › Figure S1/Figure S1.pdf]

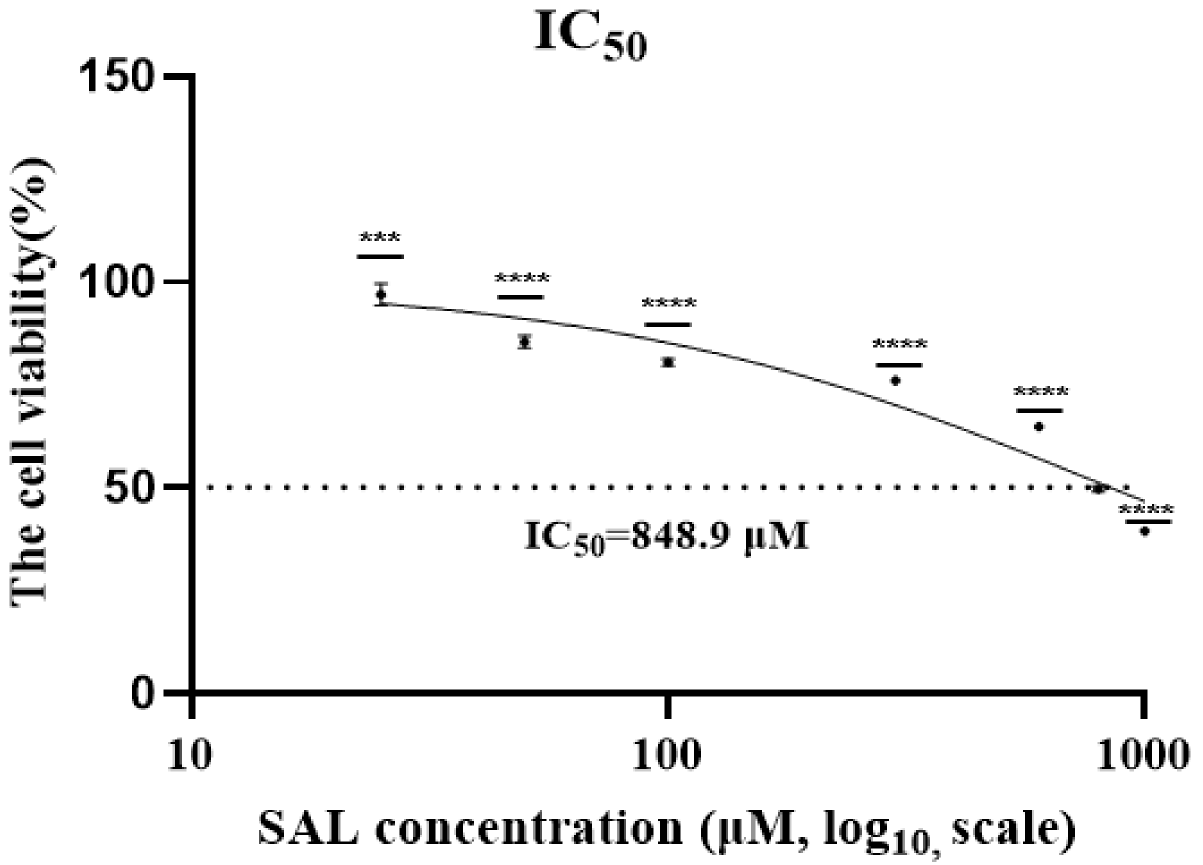

IC50
150
100
50
0
The cell viability(%)
***
****
****
****
****
****
IC50=848.9 μM
10
100
1000
SAL concentration (μM, log10, scale)
