# Supplementary material for: Salidroside Represses the Viability of Mouse Sertoli Cell Line (TM4 Cells) via Repression of Ldha-Dependent Glycolysis
Source: Molecules. 2026 Oct 8;31(19):3575. doi: 10.3390/molecules31193575 (PMC13666587; doi:10.3390/molecules31193575)
Supplement: Supplementary file 1 [file molecules-31-03575-s001.zip › Figure S2/Figure S2.pdf]

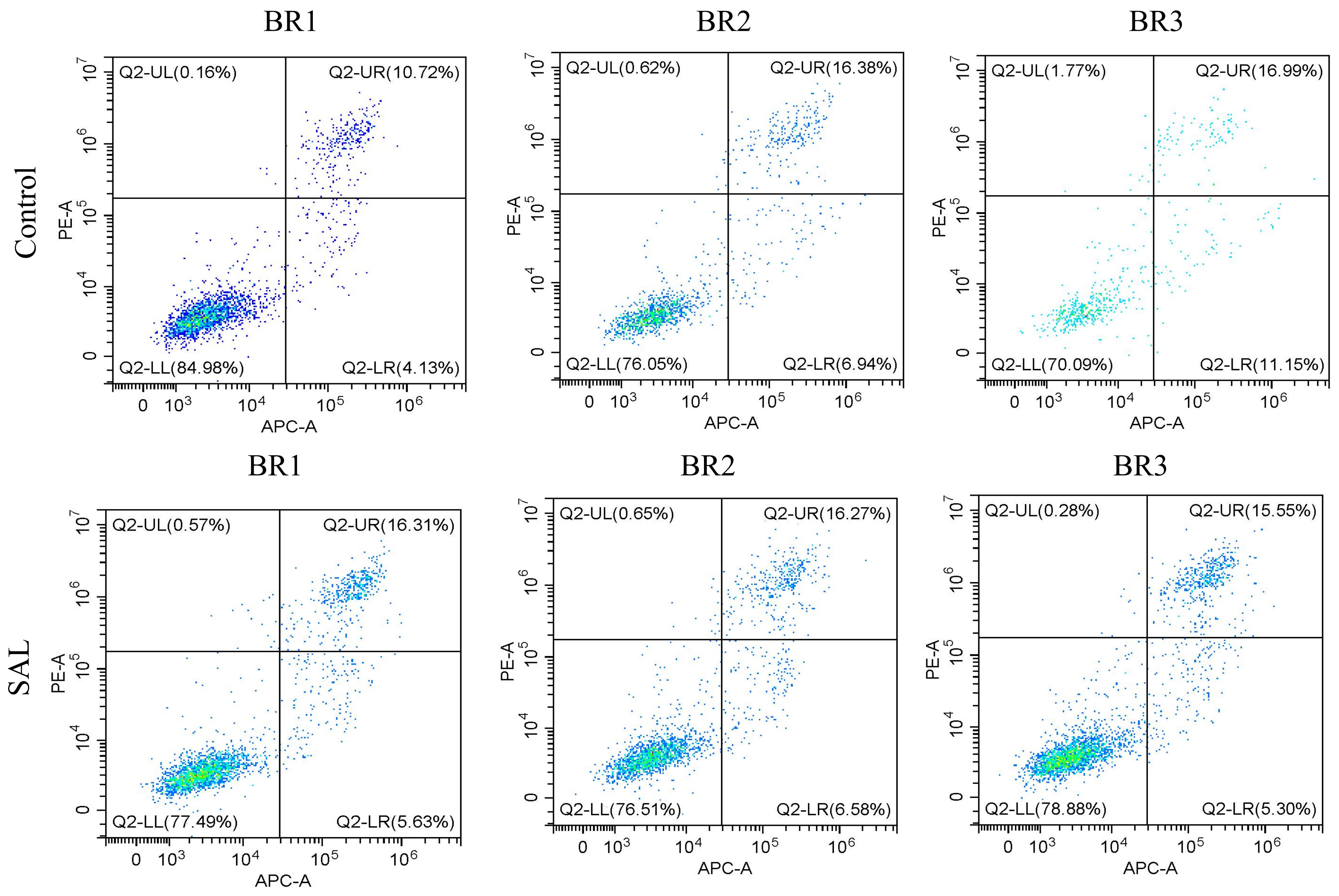

BR1
BR2
BR3
Control
Q2-UL(0.16%)
Q2-UR(10.72%)
Q2-LL(84.98%)
Q2-LR(4.13%)
Q2-UL(0.62%)
Q2-UR(16.38%)
Q2-LL(76.05%)
Q2-LR(6.94%)
Q2-UL(1.77%)
Q2-UR(16.99%)
Q2-LL(70.09%)
Q2-LR(11.15%)
SAL
Q2-UL(0.57%)
Q2-UR(16.31%)
Q2-LL(77.49%)
Q2-LR(5.63%)
Q2-UL(0.65%)
Q2-UR(16.27%)
Q2-LL(76.51%)
Q2-LR(6.58%)
Q2-UL(0.28%)
Q2-UR(15.55%)
Q2-LL(78.88%)
Q2-LR(5.30%)
PE-A
APC-A
