# Supplementary material for: Salidroside Represses the Viability of Mouse Sertoli Cell Line (TM4 Cells) via Repression of Ldha-Dependent Glycolysis
Source: Molecules. 2026 Oct 8;31(19):3575. doi: 10.3390/molecules31193575 (PMC13666587; doi:10.3390/molecules31193575)
Supplement: Supplementary file 1 [file molecules-31-03575-s001.zip › Figure S3/Figure S3.pdf]

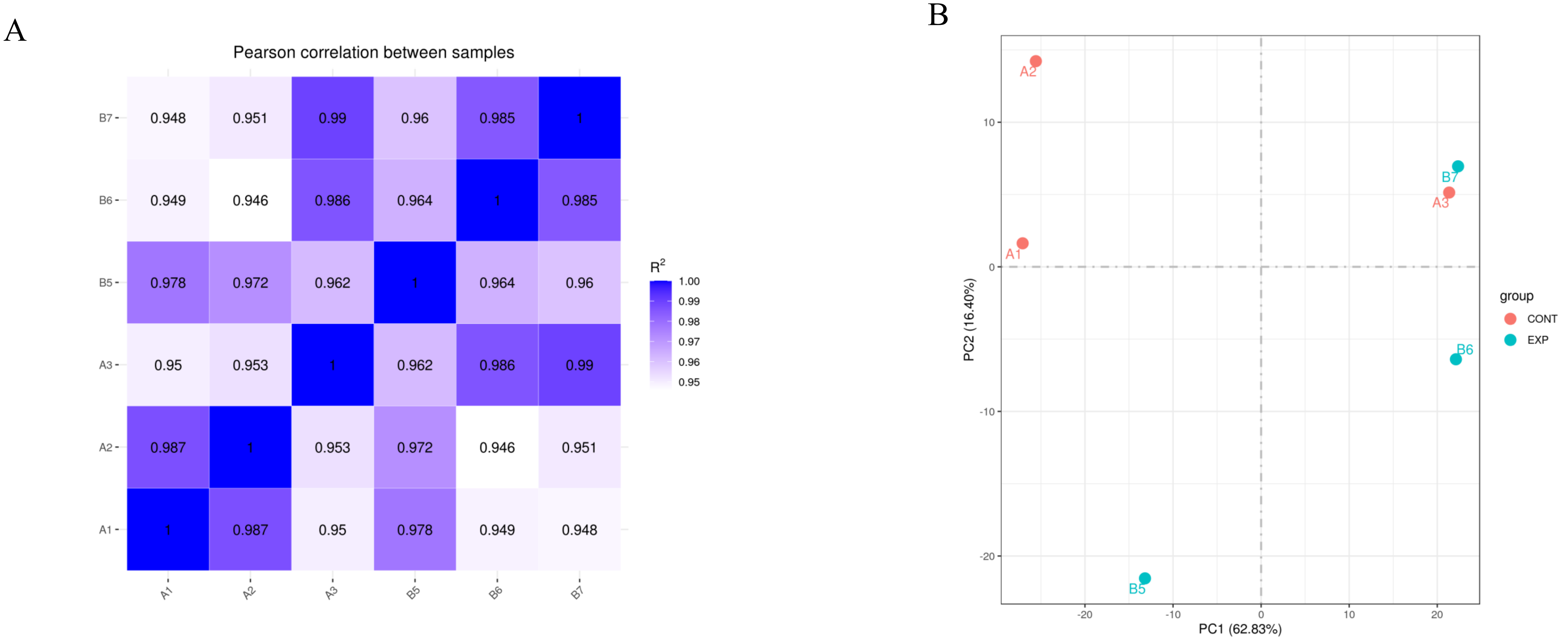
A
Pearson correlation between samples
B7 0.948 0.951 0.99 0.96 0.985 1
B6 0.949 0.946 0.986 0.964 1 0.985
B5 0.978 0.972 0.962 1 0.964 0.96
A3 0.95 0.953 1 0.962 0.986 0.99
A2 0.987 1 0.953 0.972 0.946 0.951
A1 1 0.987 0.95 0.978 0.949 0.948
A1 A2 A3 B5 B6 B7
R2
1.00
0.99
0.98
0.97
0.96
0.95
B
A2
B7
A3
A1
B6
B5
10
0
-10
-20
-20
-10
0
10
20
PC2 (16.40%)
PC1 (62.83%)
group
CONT
EXP
