# Supplementary material for: Salidroside Represses the Viability of Mouse Sertoli Cell Line (TM4 Cells) via Repression of Ldha-Dependent Glycolysis
Source: Molecules. 2026 Oct 8;31(19):3575. doi: 10.3390/molecules31193575 (PMC13666587; doi:10.3390/molecules31193575)
Supplement: Supplementary file 1 [file molecules-31-03575-s001.zip › Figure S4/Figure S4.pdf]

A

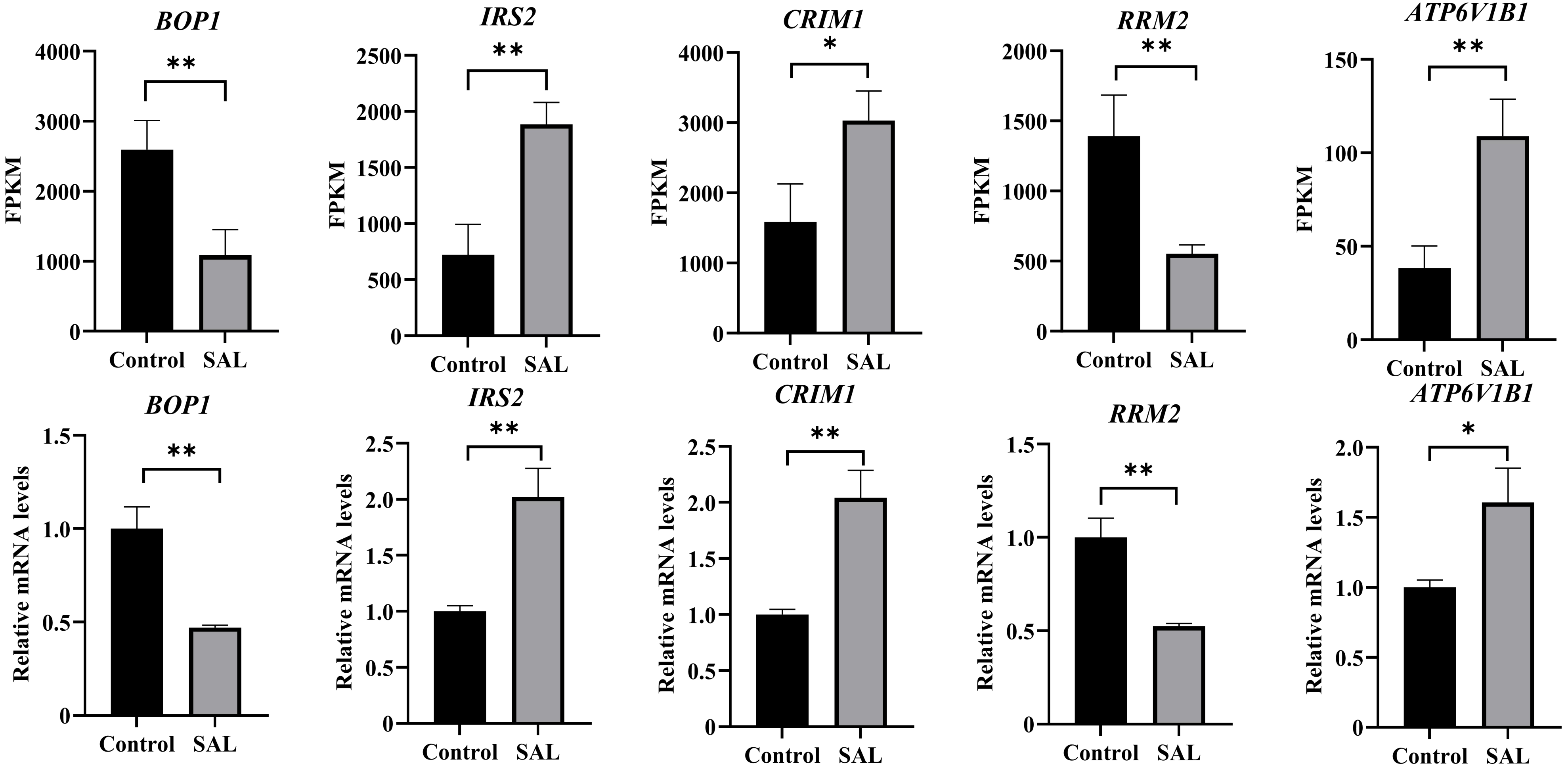

BOP1
IRS2
CRIM1
RRM2
ATP6V1B1
FPKM
Relative mRNA levels
Control
SAL
**
*
