# Supplementary material for: Salidroside Represses the Viability of Mouse Sertoli Cell Line (TM4 Cells) via Repression of Ldha-Dependent Glycolysis
Source: Molecules. 2026 Oct 8;31(19):3575. doi: 10.3390/molecules31193575 (PMC13666587; doi:10.3390/molecules31193575)
Supplement: Supplementary file 1 [file molecules-31-03575-s001.zip › Figure S5/Figure S5.pdf]

A

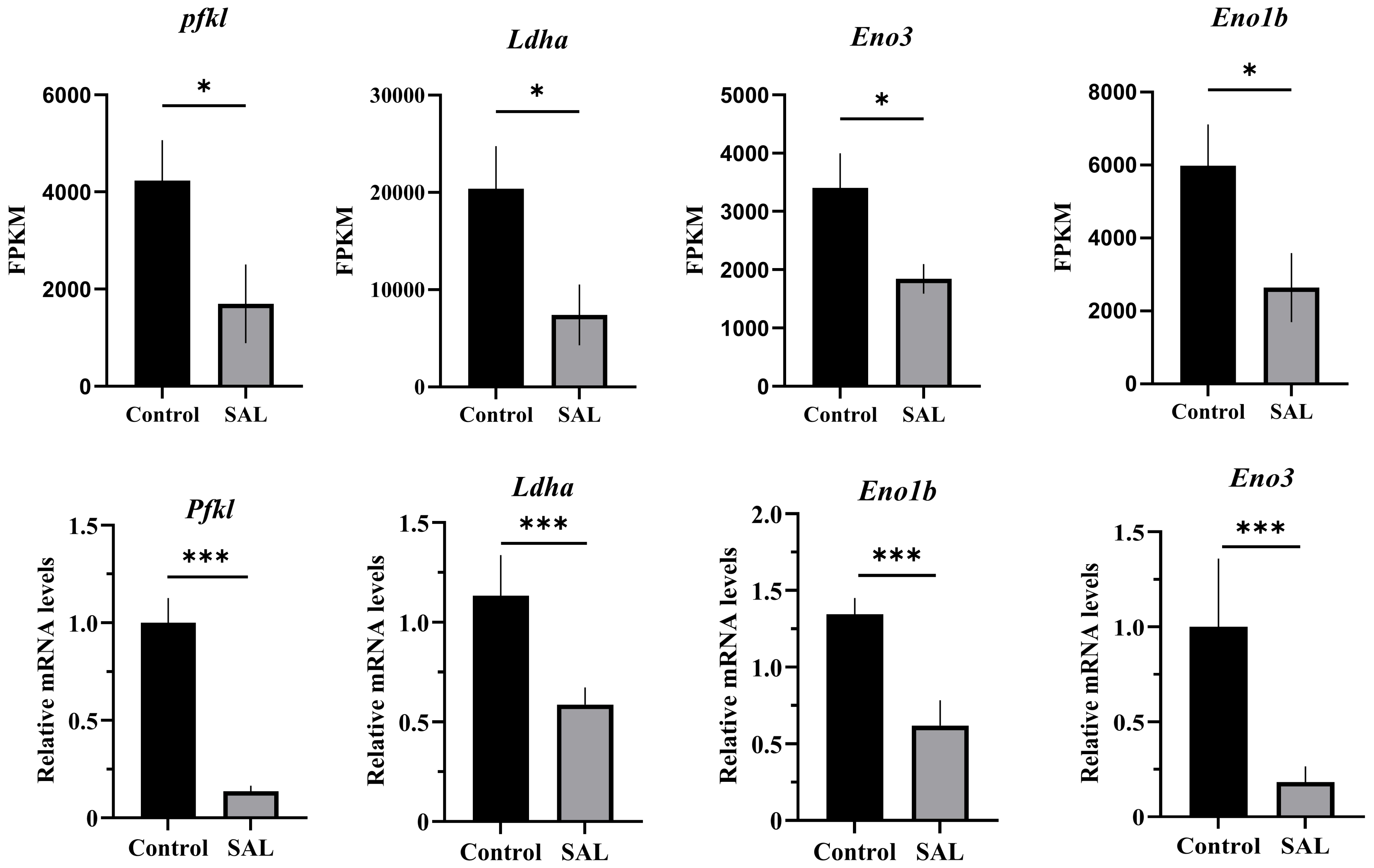

pfkl
Ldha
Eno3
Eno1b
FPKM
*
6000
4000
2000
0
30000
20000
10000
5000
3000
1000
8000
Control
SAL
Pfkl
***
Relative mRNA levels
1.5
1.0
0.5
2.0
