# Supplementary material for: Salidroside Represses the Viability of Mouse Sertoli Cell Line (TM4 Cells) via Repression of Ldha-Dependent Glycolysis
Source: Molecules. 2026 Oct 8;31(19):3575. doi: 10.3390/molecules31193575 (PMC13666587; doi:10.3390/molecules31193575)
Supplement: Supplementary file 1 [file molecules-31-03575-s001.zip › Figure S6/Figure S6.pdf]

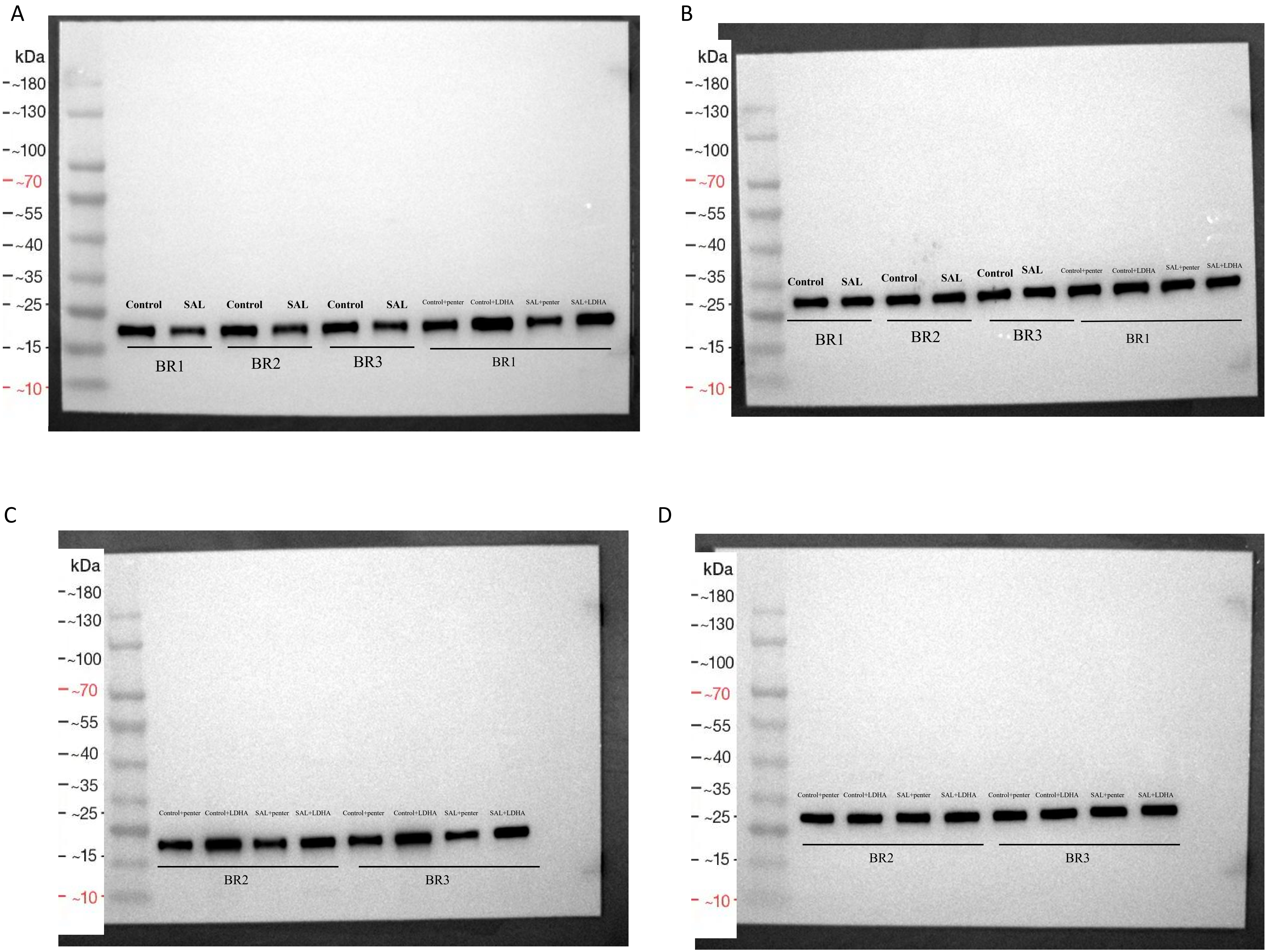
A
kDa
~180
~130
~100
~70
~55
~40
~35
~25
~15
~10
Control
SAL
Control
SAL
Control
SAL
Control+penter
Control+LDHA
SAL+penter
SAL+LDHA
BR1
BR2
BR3
BR1
B
kDa
~180
~130
~100
~70
~55
~40
~35
~25
~15
~10
Control
SAL
Control
SAL
Control
SAL
Control+penter
Control+LDHA
SAL+penter
SAL+LDHA
BR1
BR2
BR3
BR1
C
kDa
~180
~130
~100
~70
~55
~40
~35
~25
~15
~10
Control+penter
Control+LDHA
SAL+penter
SAL+LDHA
Control+penter
Control+LDHA
SAL+penter
SAL+LDHA
BR2
BR3
D
kDa
~180
~130
~100
~70
~55
~40
~35
~25
~15
~10
Control+penter
Control+LDHA
SAL+penter
SAL+LDHA
Control+penter
Control+LDHA
SAL+penter
SAL+LDHA
BR2
BR3
