# Supplementary material for: Salidroside Represses the Viability of Mouse Sertoli Cell Line (TM4 Cells) via Repression of Ldha-Dependent Glycolysis
Source: Molecules. 2026 Oct 8;31(19):3575. doi: 10.3390/molecules31193575 (PMC13666587; doi:10.3390/molecules31193575)
Supplement: Supplementary file 1 [file molecules-31-03575-s001.zip › Figure S7/Figure S7.pdf]

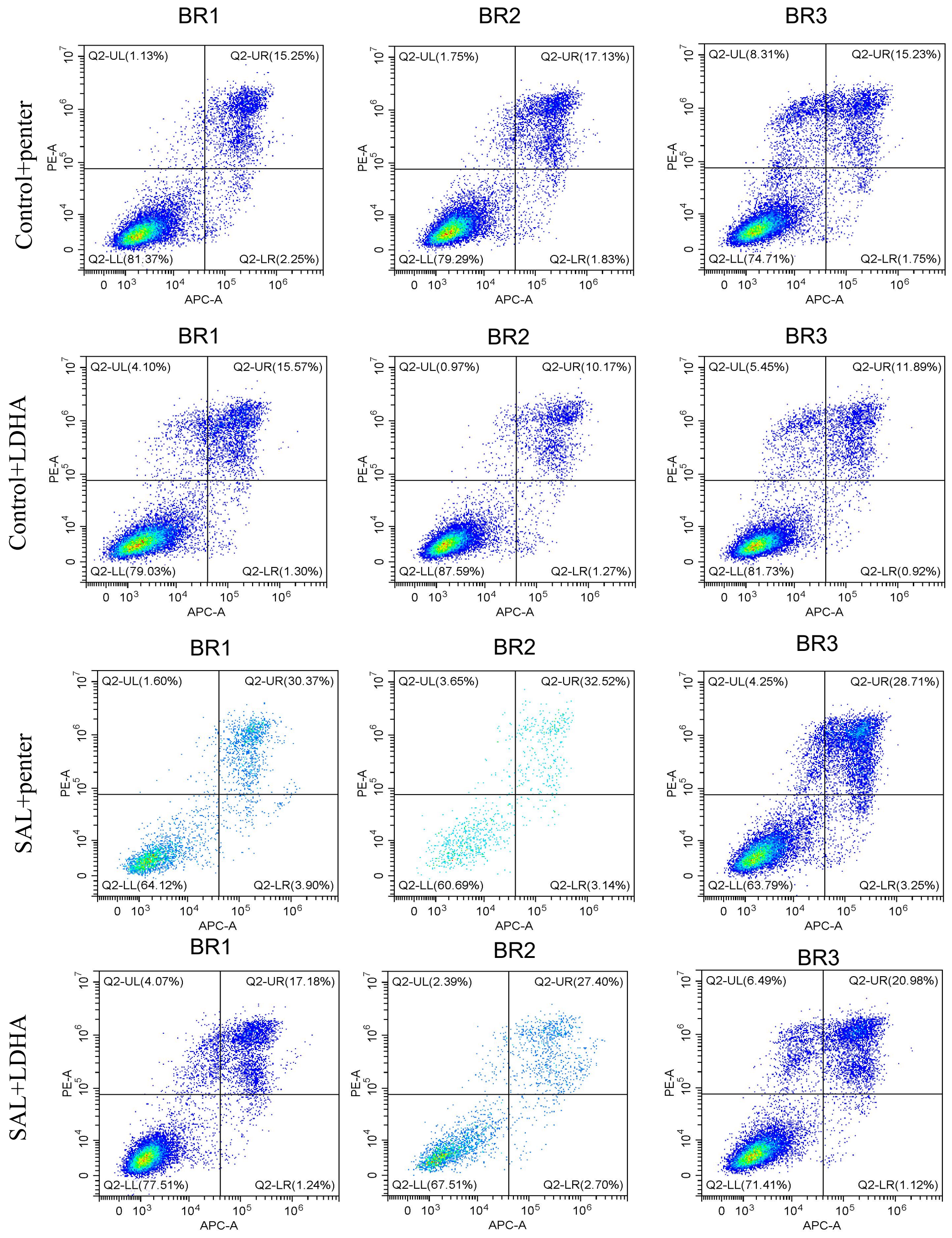

Control+penter
Control+LDHA
SAL+penter
SAL+LDHA
BR1
BR2
BR3
Q2-UL(1.13%)
Q2-UR(15.25%)
Q2-LL(81.37%)
Q2-LR(2.25%)
Q2-UL(1.75%)
Q2-UR(17.13%)
Q2-LL(79.29%)
Q2-LR(1.83%)
Q2-UL(8.31%)
Q2-UR(15.23%)
Q2-LL(74.71%)
Q2-LR(1.75%)
Q2-UL(4.10%)
Q2-UR(15.57%)
Q2-LL(79.03%)
Q2-LR(1.30%)
Q2-UL(0.97%)
Q2-UR(10.17%)
Q2-LL(87.59%)
Q2-LR(1.27%)
Q2-UL(5.45%)
Q2-UR(11.89%)
Q2-LL(81.73%)
Q2-LR(0.92%)
Q2-UL(1.60%)
Q2-UR(30.37%)
Q2-LL(64.12%)
Q2-LR(3.90%)
Q2-UL(3.65%)
Q2-UR(32.52%)
Q2-LL(60.69%)
Q2-LR(3.14%)
Q2-UL(4.25%)
Q2-UR(28.71%)
Q2-LL(63.79%)
Q2-LR(3.25%)
Q2-UL(4.07%)
Q2-UR(17.18%)
Q2-LL(77.51%)
Q2-LR(1.24%)
Q2-UL(2.39%)
Q2-UR(27.40%)
Q2-LL(67.51%)
Q2-LR(2.70%)
Q2-UL(6.49%)
Q2-UR(20.98%)
Q2-LL(71.41%)
Q2-LR(1.12%)
PE-A
APC-A
